# QuickStats

**Published:** 2014-11-14

**Authors:** FROM THE NATIONAL CENTER FOR HEALTH STATISTICS

**Figure:**
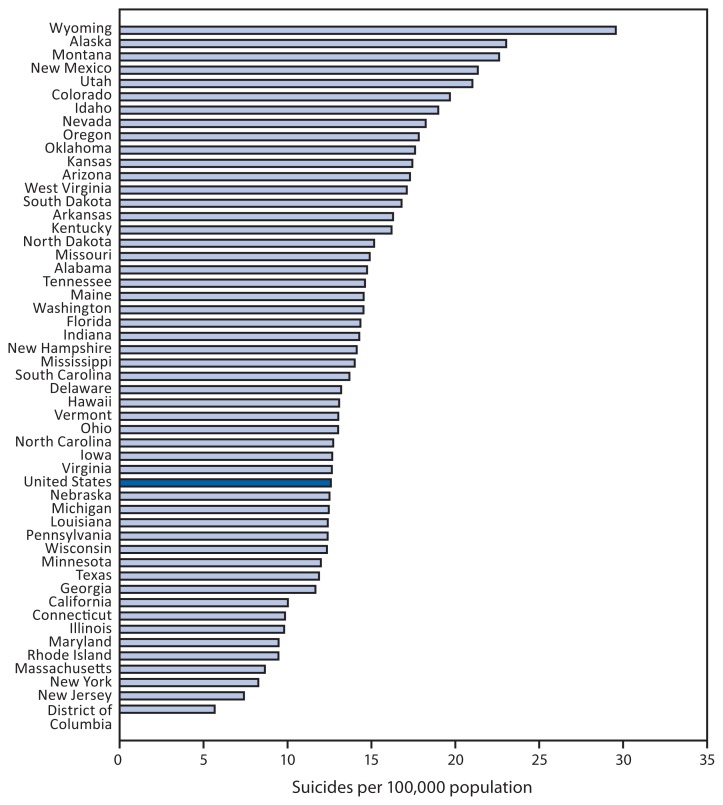
Age-Adjusted* Suicide^†^ Rates, by State^§^ — United States, 2012 * Age-adjusted rates per 100,000 based on the 2000 U.S. standard population. Populations used for computing death rates are postcensal estimates based on the 2010 census estimated as of July 1, 2012. ^†^ Intentional self-harm (suicide) as the underlying cause of death includes codes for by discharge of firearms (X72–X74), and Intentional self-harm (suicide) by other and unspecified means and their sequelae (U03,X60–X71,X75–X84,Y87.0), in the *International Classification of Diseases, 10th Revision*. ^§^ U.S. residents only.

In 2012, the overall age-adjusted suicide rate in the United States was 12.6 per 100,000 population. Among states, Wyoming had the highest suicide rate (29.6), followed by Alaska (23.0), Montana (22.6), New Mexico (21.3), and Utah (21.0). The District of Columbia had the lowest suicide rate (5.7), followed by New Jersey (7.4), New York (8.3), Massachusetts (8.7), and Rhode Island (9.5). For 34 states, suicide rates were higher than the overall U.S. rate. In 2012, a total of 40,600 suicides were reported in the United States.

**Source:** National Vital Statistics System. Mortality public use data files, 2012. Available at http://www.cdc.gov/nchs/data_access/vitalstatsonline.htm.

**Reported by:** Betzaida Tejada-Vera, MS, fsz2@cdc.gov, 301-458-4231.

